# Real-world use of Serial Clinical Observation in culture-proven early-onset sepsis: timing of recognition, treatment and retrospective comparison with the Neonatal Sepsis Calculator

**DOI:** 10.1007/s00431-026-06905-7

**Published:** 2026-04-16

**Authors:** Francesca Miselli, Licia Lugli, Giorgia Dragone, Luca Bedetti, Sofia Spinedi, Mariagrazia Capretti, Lucia Marrozzini, Luisa Di Luca, Silvia Fanaro, Lucia Gambini, Giacomo Biasucci, Giancarlo Piccinini, Davide Scarponi, Irene Papa, Francesca Nanni, Lorenza Baroni, Rossella Pagano, Valeria Capone, Alberto Berardi

**Affiliations:** 1 Neonatal Intensive Care Unit, University Hospital of Modena, Via del Pozzo, 41124 Modena, Italy; 2 Postgraduate Training in Obstetrics and Gynecology, University of Torino, Via Verdi 8, 10124 Turin, Italy; 3 Maggiore University Hospital, Largo Bartolo Nigrisoli 2, 40133 Bologna, Italy; 4 S. Orsola-Malpighi University Hospital, Via Giuseppe Massarenti 9, 40138 Bologna, Italy; 5 Ramazzini Hospital of Carpi, Via Giovanni Rodolfo Baroni 1, 41012 Carpi, Modena Italy; 6 Bufalini Hospital, Viale Ghirotti 286, 47521 Cesena, Italy; 7 Cona University Hospital, Via Aldo Moro 8, 44124 Cona, Ferrara Italy; 8 University Hospital, Via Gramsci 14, 43126 Parma, Italy; 9 Guglielmo da Saliceto Hospital, Via Taverna 49, 29121 Piacenza, Italy; 10 S. Maria Delle Croci Hospital, Viale Randi 5, 48121 Ravenna, Italy; 11 Infermi Hospital, Viale Luigi Settembrini 2, 47923 Rimini, Italy; 12 Santa Maria Nuova Hospital, Viale Risorgimento 80, 42123 Reggio Emilia, Italy; 13 Hospital of Sassuolo, Via Francesco Ruini 2, 41049 Sassuolo, Modena Italy; 14 PhD Program in Clinical and Experimental Medicine, University of Modena and Reggio Emilia, Via Università 4, 41121 Modena, Italy

**Keywords:** Early-onset sepsis, Serial clinical observation, Serial physical examination, Neonatal sepsis calculator, Sepsis risk calculator

## Abstract

To assess the real-world performance of the Serial Clinical Observation (SCO) strategy in identifying and treating Early-Onset Sepsis (EOS) cases, and to compare it with the recommendations the Neonatal Sepsis Calculator (NSC) would have provided. This multicentre study analyzed prospectively culture-confirmed EOS cases caused by *Group B Streptococcus* (GBS) or *Escherichia coli* (2016–2022) in term and late-preterm infants managed with the SCO approach in Italy. We evaluated the proportion of infants assigned to routine care at birth and the time to antibiotics. NSC scores at 4, 12, and 24 h after birth were retrospectively calculated. Of 57 EOS cases, 23 (40.4%) had no maternal risk factors. Fifty infants developed clinical signs of illness either at birth (*n* = 24) or postnatally (*n* = 26). Among those symptomatic at birth, the median time to antibiotic initiation was 2.0 h (IQR 1.0–5.75). “Major” clinical signs according to the SCO protocol were present in 79.2%, prompting immediate antibiotics. Retrospective NSC application at 4 h would have recommended antibiotics in 87.5%. Of the 26 who became symptomatic after birth, 16 (61.5%) were assigned to routine care. The median time from disease onset to treatment was 6.0 h (IQR 2.0–14.0). At 4 h, NSC would have assigned 19 (73.1%) of these infants to routine care. Subtle neurological signs (i.e. hypotonia, irritability, altered consciousness) were frequently observed at onset in infants who developed severe disease.

*Conclusion*: Most EOS cases were appropriately identified among infants with clinical signs at birth, but not among those initially well-appearing. Close clinical observation throughout the first 48 h of life, along with careful attention to “major” signs—including subtle neurological signs—may improve EOS management.

**Table:** 

**What is Known:** • *The Serial Clinical Observation (SCO) strategy reduces unnecessary antibiotic use and laboratory testing in infants at risk of Early-Onset Sepsis (EOS).* • *However, its performance in identifying confirmed EOS cases has never been established nor compared with that of the Neonatal Sepsis Calculator (NSC).*
**What is New:** • *Among neonates clinically ill at birth, SCO and NSC recommended immediate antibiotics in a similar proportion (79% vs 88%).* • *Among infants with EOS who were initially well-appearing, most were assigned to routine care under the SCO approach (62%) or would have been assigned to routine care under the NSC approach (73%).* • *To ensure timely recognition of EOS, universal observation of all infants over the first 24–48 h after birth may be beneficial.*

**Supplementary Information:**

The online version contains supplementary material available at 10.1007/s00431-026-06905-7.

## Introduction

Early-onset sepsis (EOS, occurring from 0 to 72 h of life) remains a potentially fatal neonatal complication. Over recent decades, its incidence has significantly declined [1–5], largely due to effective obstetric measures and the implementation of intrapartum antibiotic prophylaxis (IAP) for Group B Streptococcus (GBS) [1, 6]. However, the initial clinical signs of EOS are often nonspecific, and current laboratory tests lack the sensitivity and specificity required to reliably guide management decisions. Current approaches to managing neonates at risk of EOS remain controversial [7–9]. On one side, delayed antibiotic treatment can have devastating consequences. On the other, for each confirmed case of EOS, more than 100–200 neonates receive empirical antibiotics [10–12], raising concerns about the long-term adverse effects of early antibiotic exposure [13, 14].

The Neonatal Sepsis Risk Calculator (NSC) is a multivariate risk assessment tool increasingly utilized worldwide for managing neonates at risk of EOS, combining the local incidence of EOS and maternal risk factors (pretest probability) with the evolving clinical condition of the newborn during the first hours of life [5, 15–17]. By contrast, the Serial Clinical Observation (SCO) is a risk assessment strategy primarily based on the clinical condition of infants at risk, from birth to 48 h of age [18–20]. Both are widely used strategies recommended by the American Academy of Pediatrics (AAP) for managing term and late-preterm neonates at risk of EOS [8]. Compared to the categorical approach (i.e. the third management strategy recommended by the AAP for EOS), both NSC and SCO have reduced unnecessary antibiotic exposure in uninfected neonates [16, 18, 21, 22]. However, it remains unclear how promptly these two strategies identify and treat neonates with EOS. A meta-analysis showed that the NSC recommends initiation of antibiotics at birth in 40% of culture-proven EOS cases [23]. No studies have examined how promptly antibiotics are administered to newborns with culture-proven EOS under the SCO strategy. It has been argued that large-scale studies, ideally at the population level, are required before broad adoption to confirm the safety of SCO in different settings internationally [5]. The aim of this study was to assess the real-world performance of the SCO strategy through an Italian region-wide prospective EOS surveillance network. Among culture-confirmed GBS and *E. coli* EOS cases in infants ≥ 34 weeks’ gestation, we evaluated (1) the proportion of infants assigned to routine care at birth and (2) the observed delays to recognition (i.e., blood culture collection) and treatment under the SCO strategy. As a secondary analysis, we retrospectively calculated the NSC recommendations for these infants, comparing initial risk stratification between the two approaches.

## Methods

### Study design

This was a multicenter study based on a pre-existing Italian prospective, area-based surveillance network specifically designed to monitor EOS caused by GBS and *Escherichia coli* (*E.coli*) [3, 24]. Infants with a gestational age ≥ 34 weeks’ gestation who developed EOS due to either of these two pathogens (January 1 st, 2016–December 31 st, 2022) were included. The study was carried out in Emilia-Romagna, a region with approximately 35,000 live births (LBs) per year and 22 birthing centres. All centres implement a universal screening-based strategy to prevent GBS EOS [25, 26] and follow the SCO protocol to manage infants at risk of EOS [27]. The primary aim of this study was to prospectively describe the real-world performance of the SCO strategy in culture-confirmed EOS cases, evaluating (1) the proportion of infants who were initially assigned to routine care and (2) the timing of symptom recognition (i.e. blood culture collection) and first antibiotic administration. As a secondary analysis, we retrospectively calculated the NSC scores at 4, 12, and 24 h after birth, to evaluate provided recommendations. Local investigators extracted data from clinical records: demographics, antenatal risk factors, presence of 22 clinical signs at disease onset (Online Resource 1), investigations, and antibiotics. To maintain patient confidentiality, spreadsheets submitted to the principal investigator were anonymous. Any missing information was resolved through phone contact with local investigators. The project was approved by the ethics committee (protocol no.910/2020/OSS/AOUMO). The Strengthening the Reporting of Observational Studies in Epidemiology (STROBE) reporting guidelines were followed for this study.

### Definitions

#### Early-onset sepsis

GBS or *E. coli* isolated from blood and/or cerebrospinal fluid from 0 to 72 h of life [8].

#### Neonates at risk

neonates delivered by a mother colonized with GBS or with risk factors for EOS: prolonged rupture of membranes (≥ 18 h); intrapartum maternal fever (≥ 38.0 °C); GBS bacteriuria; previous infant with invasive GBS disease [8].

#### Disease onset

the earliest time at which signs of illness were recognized by healthcare staff, as recorded in the clinical chart.

#### Severe disease 

presence of any of the following: death, meningitis, seizures, brain lesions at hospital discharge, need for catecholamine support, or mechanical ventilation [28, 29].

#### Brain injury

any brain lesion detected at neuroimaging (ultrasound and/or magnetic resonance imaging) described in the literature as associated with neonatal infections, including stroke-like lesions, ventriculitis, hydrocephalus, meningeal adhesions, haemorrhagic necrosis, and intraventricular haemorrhage [30].

#### “Major” clinical signs

any of the following: moderate-to-severe respiratory distress requiring respiratory support (i.e. need for positive pressure ventilation outside of the delivery room); hypoxia (i.e., need for supplemental O_2_ to maintain oxygen saturations > 90% outside of the delivery room); reduced skin perfusion; refill ≥ 3 s; signs of shock; temperature ≥ 38 °C (outside of the delivery room); worsening of the general condition; apnoea; lethargy; irritability; seizures; greyish, pallor, or marbling of the skin colour [31].

### Management of neonates in the study cohort according to the Serial Clinical Observation protocol

In our network, the SCO protocol is applied to infants ≥ 34 weeks’ gestation at risk, who undergo a structured program of standardized serial clinical assessments over the first 48 h of life [19]. For serial clinical assessments, a standardized form is used detailing the general wellbeing (yes/no), skin colour (normal or pink/pale/mottled/cyanotic), and respiratory signs (i.e. respiratory distress/chest retractions/tachypnoea) at predefined intervals (1, 3, 6, 12, 18, 24, 36, and 48 h of life). Intervals are scheduled at closer succession during the initial hours of life, based on our previous studies indicating that the majority of EOS cases manifest within this critical early period [29]. The standardized form is signed by the examiner (physician, trained nurse, or midwife) and included in the neonate’s medical records. Infants are allowed to room-in with their own mother, and serial clinical assessments are usually conducted in a rooming-in setting [31]. There is no specific clinical evaluation protocol for neonates considered “not at risk” and assigned to routine care. However, during skin-to-skin contact after birth, midwives assess all newborns approximately every 15–30 min, using the same clinical parameters adopted in the SCO evaluations, for up to 2 h of life. Under routine care, a medical examination is performed within 4–6 h after birth and before home discharge.

Newborns who develop clinical signs suggestive of sepsis are promptly referred to a neonatal care specialist. Their signs are further classified in more detail (as “minor” and “major”) to guide clinical management. “Major” signs trigger immediate blood culture and initiation of empirical antibiotics (Online Resource 2) [27]. By contrast, at the clinician’s discretion, neonates presenting with mild and non-progressive signs—potentially attributable to non-infectious conditions typical of the transitional period—may be re-evaluated at 2-h intervals, withholding laboratory evaluations and antibiotics. If these minor signs persist or worsen, blood culture is collected and empirical antibiotics started. After implementing this SCO approach, overall antibiotic exposure is low (1.7%) in our region [11, 31].

### Neonatal sepsis calculator recommendations

For each infant included in the study, the NSC scores at 4, 12, and 24 h after birth were retrospectively assigned based on a review of medical records (conducted between September 2023 and June 2024). Two researchers (G.D. and A.B.) independently calculated the scores using the online NSC tool (neonatalsepsiscalculator.kaiserpermanente.org). The assumed EOS incidence rate (0.6 per 1000 LBs) was derived from our previous local data [24] and from the developmental set of the NSC, as it has been suggested that the NSC performs best when the incidence from the developmental set is used, independently of the true EOS incidence in a particular population [32]. Since the updated version of the NSC [33] was not available at the time of analysis, we calculated the scores based on the previously published version [17].

#### Statistical analyses

We used MedCalc version 9.3. Continuous variables were expressed as median and interquartile range (IQR); categorical data were presented as numbers (percentages). The 95% confidence intervals (95% CI) for proportions were calculated using the Wald method. Comparisons between patient groups were performed using the chi-square test, Fisher’s exact test, Student’s *t*-test, or Mann–Whitney test, as appropriate. All *p*-values < 0.05 were considered significant.

## Results

From 2016 to 2022, 223,275 LBs occurred (of which 219,146 at ≥ 34 weeks’ gestation). Among these, 58 neonates were diagnosed with GBS or *E. coli* EOS (0.26 per 1000 LBs, 95% CI 0.20–0.33 per 1000 LBs). The medical record of one neonate was unavailable, leaving 57 neonates for analysis (term neonates, *n* = 49; late-preterm neonates, *n* = 8; GBS, *n* = 33;* E. coli*, *n* = 24).

### Overall cohort

Demographics and clinical characteristics of the study population are detailed in Table 1. Overall, 40.4% of neonates with EOS did not have any maternal risk factors. Fifty out of 57 neonates (87.7%) developed clinical signs of illness prior to discharge (median age at disease onset 1 h, IQR 0–9) and two died. In contrast, seven neonates (12.3%) remained asymptomatic (blood cultures were collected due to maternal risk factors). Among these, three neonates received antibiotics from birth, three after blood culture results, and one remained untreated.

**Table 1:** Demographics and clinical characteristics of the study population (57 cases of Early-Onset Sepsis)

Variables	All (*n* = 57)
Birth weight, median, g (IQR)	3160 (2815–3625)
Gestational age at delivery, median, weeks (IQR)	39 (38.0–40.0)
Intrapartum fever ≥ 38 °C, *n* (%)^‡^	13 (22.8)
ROM ≥ 18 h, *n* (%)	18 (31.6)
Prenatal screening, *n* (%)	51 (89.5)
Positive prenatal screening, *n* (%)^§^	14 (27.5)
No RFs, GBS screening negative or unknown, *n* (%)	23 (40.4)
IAP exposure, *n* (%)	27 (47.4)
Antibiotics ≥ 4 h, *n* (%)^¶^	9 (33.3)
Antibiotics < 2 h, *n* (%)^¶^	11 (40.7)
Never developed signs of illness, *n* (%)^¥^	7 (12.3)
Clinical signs of illness at birth, *n* (%)^*^	24 (48.0)
Developed clinical signs at 1 to 24 h of life, *n* (%)^*^	18 (36.0)
Developed clinical signs after 24 h of life, *n* (%)^*^	8 (16.0)
Hours from onset of clinical signs to blood culture, median, hours (IQR)	1.0 (0.0–5.0)
Antibiotics given, *n* (%)	56 (98.2)
Hours from blood culture to antibiotics, median, (IQR)	0 (0.0–3.0)
Mechanical ventilation, *n* (%)	7 (12.3)
Catecholamines, *n* (%)	6 (10.5)
Meningitis (± sepsis), *n* (%)^†^	5 (8.8)
Brain lesions, *n* (%)	2 (3.5)
Severe disease, *n* (%)	15 (26.3)
Case fatalities, *n* (%)	2 (3.5)

*GBS *group B streptococcus, *IAP *intrapartum antibiotic prophylaxis, *RFs *risk factors, *ROM* rupture of membranes

^‡^In five cases, maternal temperature was not recorded in the medical chart, while in six cases, it was documented as “no fever” without specifying the exact value. For all these cases, we calculated the score using a temperature of 37 °C

^§^Percentage is calculated among women who underwent antenatal vagino-rectal screening

^¶^Percentages are calculated among cases exposed to intrapartum antibiotic prophylaxis. Ampicillin was used for intrapartum prophylaxis in all cases except one, in which clindamycin was administered

^*^Percentages are calculated among the 50 infants who became symptomatic. Asymptomatic cases are excluded

^¥^One out of seven infants was never treated with antibiotics

^†^Meningitis: clinical signs of meningitis and positive cerebrospinal fluid culture (or *PCR* test). Seven neonates (2 with GBS and 5 with *E. coli* infection) received no lumbar puncture

**Fig. 1:**
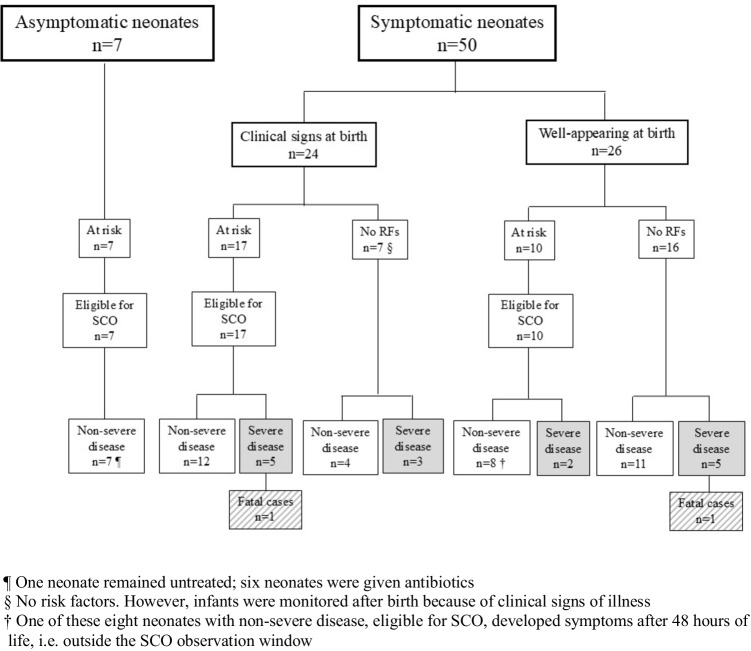
Eligibility for the Serial Clinical Observation (SCO) protocol in the study cohort (57 infants with early-onset sepsis) according to the presence of clinical signs and/or risk factors (RFs) for early-onset sepsis and severity of their illness

**Fig. 2:**
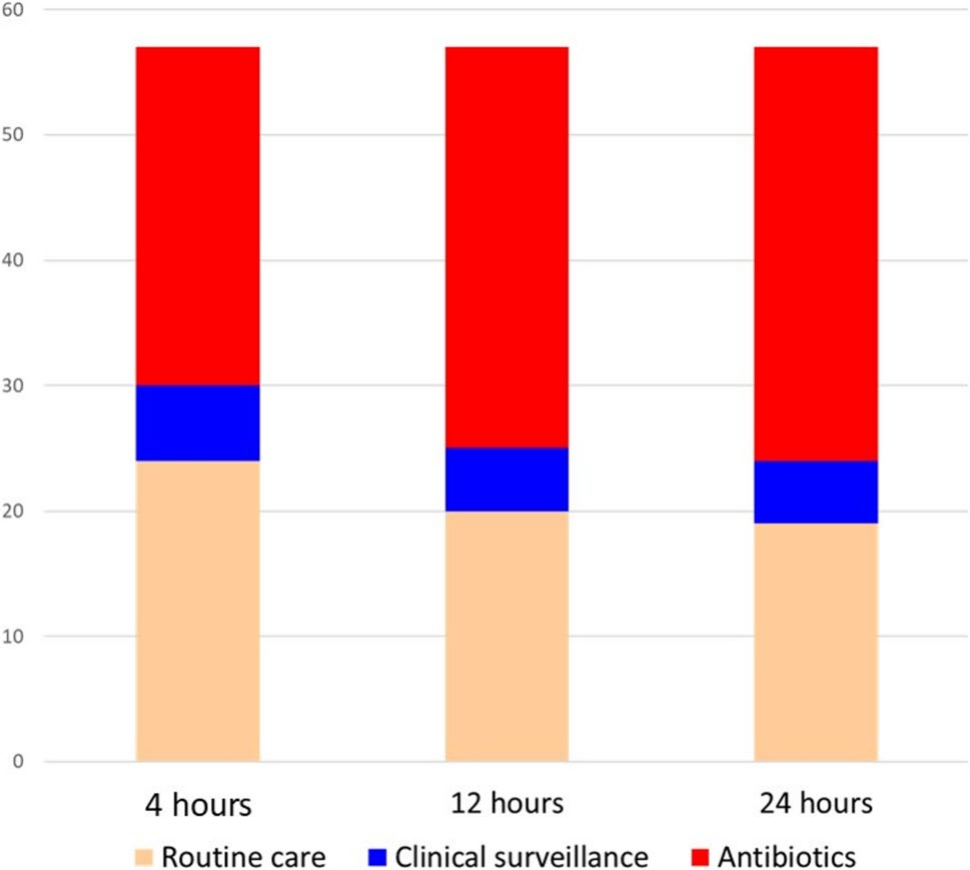
Management that would have been recommended at 4, 12, and 24 h of life in the study cohort (57 infants with Early-Onset Sepsis) according to the Neonatal Sepsis Calculator. Newborns who would have been directed to antibiotic treatment were 27 at 4 h of life (47.4%, 95% CI 34.4–60.3%); 32 at 12 h (56.1%, 95% CI 43.3–69.0%); and 33 at 24 h (57.9%, 95% CI 45.1–70.7%). Those who would have been managed with clinical monitoring were 6 at 4 h of life (10.5%, 95% CI 2.6–18.5%); 5 at 12 h (8.8%, 95% CI 1.4–16.1%); and 5 at 24 h (8.8%, 95% CI 1.4–16.1%)

Figure 1 details the eligibility for the SCO program in the 57 infants of the study cohort, according to the presence of clinical signs and/or risk factors, and severity of their illness. Had we applied the NSC, the median risk score would have been 0.33 (IQR 0.04–2.22, range 0.0–65.1). Figure 2 illustrates the neonatal management that would have been recommended according to the NSC at 4, 12, and 24 h after birth. The proportion of neonates with an indication for antibiotic administration increases progressively over the first 24 h after birth.

### Neonates who were clinically ill at birth

Among the 50 infants who developed clinical signs of illness, 24 (48.0%) were clinically ill at birth (Fig. 1). The median time to blood culture collection was 1.0 h (IQR 0.0–5.0), while the median time to first antibiotics was 2.0 h (IQR 1.0–5.75). Among these 24 neonates, 19 (79.2%, 95% CI 62.9–95.4%) presented with “major” signs at birth, triggering immediate blood culture and antibiotic treatment according to the SCO protocol. The remaining five infants (20.8%, 95% CI 4.6–35.1%) had “minor” signs and were managed according to the attending physician’s judgment. Among the 24 infants clinically ill at birth, 17 (70.8%, 95% CI 52.7–88.9%) were at risk and were eligible for the SCO program, which recommended blood culture and immediate antibiotics in 14 of them (because of “major” clinical signs) and clinical surveillance in three.

The retrospective calculation using the NSC at 4 h after birth would have recommended immediate antibiotic administration, strict clinical surveillance, and routine care for 21 (87.5%, 95% CI 74.1–100%); 2 (8.3%, 95% CI 0–19.4%); and 1 (4.2%, 95% CI 0–12.2%) neonates, respectively.

### Well-appearing neonates at birth

Among the 50 infants who developed clinical signs of illness, 26 (52.0%) were well-appearing at birth and developed initial clinical signs later on (1–24 h of life, *n* = 18; 25–48 h, *n* = 6; > 48 h, *n* = 2). The median time between disease onset and blood culture collection was 4.0 h (IQR 1.0–7.0), while the median time between disease onset and first antibiotics was 6.0 h (IQR 2.0–14.0). Of the 26 newborns who were well-appearing at birth, 16 (61.5%, 95% CI 42.8–80.2%) had no identifiable risk factors and were assigned to routine care, while 10 (38.5%, 95% CI 19.8–57.2%) were at risk for EOS and were assigned to the SCO programme (Fig. 1). One of them developed clinical signs of illness after 48 h of life, thus exceeding the SCO observation window. In four of the ten cases assigned to the SCO programme, prompt initiation of antibiotic therapy at birth was warranted due to the presence of “major” signs.

The retrospective calculation using the NSC at 4 h after birth would have recommended antibiotic administration, strict clinical surveillance, and routine care for 6 (23.1%, 95% CI 6.9–39.3%); 1 (3.9%, 95% CI 0–11.1%); and 19 (73.1%, 95% CI 56.0–90.1%) infants, respectively. Eight of 25 (30.8%) developed clinical signs of illness beyond the first 24 h of life—an age not covered by the NSC clinical monitoring window.

### Neonates with severe disease

Tables 2 and 3 detail the 15 cases of severe disease. All of these infants who developed severe disease presented with at least one “major” sign at disease onset that should have triggered immediate blood culture collection and antibiotic administration. None of these neonates initially presented with isolated respiratory clinical signs.

**Table 2:** Infants who developed severe disease and were clinically ill at birth. All presented with at least one “major” clinical sign

Cases	GA (weeks)	RFs and/or positive antenatal GBS screening	IAP † (hrs)	NSC score ^¶^	GBS/*E. coli*	Clinical signs at the onset of the disease	Age at blood culture (hours)	Age at first antibiotics (hours)	Disease severity
1	40	No	None	3.77	GBS	Tachypnoea, hypotension, hypotonia	0	0	MV, CA
2	40	No	1^‡^	2.16	*E. coli*	Tachypnoea, greyish skin colour, lethargy, hypotonia	0	5	CA
3	41	ROM, maternal fever	3	11.18	GBS	Intrapartum asphyxia, irritability, hypotonia	0	3	MV, CA
4	40	GBS +	> 4	3.88	GBS	Irritability, hypotonia, pale skin colour	5	5^††^	CA
5	38	Maternal fever	None	3.88	*E. coli*	Mottled/pale skin, reduced consciousness, lethargy, hypotonia	1	1	MV
6	38	No	None	1.88	*E. coli*	Intrapartum asphyxia, hypothermia, tachypnoea, irritability, apnoeas	5	20^§^	MV, seizures
7	36	ROM, GBS +	> 4	2.41	*E. coli*	Hypothermia, irritability, hypotonia	1	1	MV
8	34	ROM	> 4	65.1	*E. coli*	Respiratory distress, greyish skin colour, lethargy, hypotonia	1	1^¥^	MV, CA, BL, death

*BL *brain lesions, *CA *catecholamines, *GA *gestational age, *GBS *group B streptococcus, *hrs *hours, *I *inadequate intrapartum antibiotic prophylaxis, *IAP *intrapartum antibiotic prophylaxis, *MV *mechanical ventilation, *NSC *neonatal sepsis calculator, *RFs *risk factors, *ROM* rupture of membranes

^†^In all cases where intrapartum prophylaxis was administered, ampicillin was used

^¶^Neonatal sepsis risk score after clinical examination at birth

^‡^Intrapartum antibiotic prophylaxis was administered despite the duration of membrane rupture being 17 h

^††^This newborn was delivered in a level 1 center; he experienced difficulties with venous access in the first hours of life, which were resolved after the arrival of the neonatal transport team

^§^The newborn required transport to 2 different Neonatal Intensive Care Units during the first hours of life. An insufficient transmission of information between birthing centres is possible

^¥^Although the neonate had been receiving antibiotics since birth, the infection was caused by a multidrug-resistant *E. coli*. On the third day of life, the infant developed gastrointestinal complications, including severe necrotizing enterocolitis, leading to death

**Table 3:** Infants with severe disease who developed clinical signs after birth. All were well-appearing at birth and presented with at least one “major” sign at disease onset

Cases	GA (weeks)	RFs and/or positive antenatal GBS screening	IAP^†^ (hrs)	NSC score	GBS/*E. coli*	Clinical signs at the onset of the disease	Age at disease onset (hours)	Hours between symptoms and blood culture	Hours between symptoms and antibiotics	Disease severity
1	37	No	None	0.02	GBS	Tachycardia, reduced consciousness, irritability, toxic appearance, apnoeas, prolonged capillary refill time, feeding difficulties	2	5	5	Meningitis
2	40	Maternal fever	None	0.17	*E. coli*	Tachypnoea, apnoeas, feeding difficulties	4	1	1	Seizures
3	40	No	None	0.05	GBS	Poor feeding, fever, tachycardia	5	14	14	Meningitis
4	38	ROM	> 24	0.02	*E. coli*	Irritability, poor feeding, inconsolable crying	25	6^§^	11	Meningitis
5	38	No	None	0.02	GBS	Fever, tachypnoea, tachycardia, mottled/pale skin colour, irritability, feeding difficulties, jaundice	31	0	0	Meningitis, CA
6	39	No	None	0.07	*E. coli*	Toxic appearance, poor feeding, lethargic, tachypnoea, tachycardia	44	0	0	MV, BL, death
7	39	No	None	0.03	GBS	Fever, tachypnea, tachycardia, irritability, feeding difficulties	48	0	0	Meningitis

*BL *brain lesions, *CA *catecholamines, *GA *gestational age, *GBS *group B streptococcus, *hrs *hours, *IAP *intrapartum antibiotic prophylaxis, *MV *mechanical ventilation, *NSC *neonatal sepsis calculator, *RFs *risk factors, *ROM* rupture of membranes

^†^In all cases where intrapartum prophylaxis was administered, ampicillin was used

^§^This newborn was exposed to intrapartum antibiotic prophylaxis (lasting 26 h). He developed feeding difficulties at 25 h of life. At 31 h, his condition worsened, with the onset of inconsolable crying, and a blood culture was obtained. A severe clinical deterioration followed shortly thereafter. The *E. coli* strain was resistant to ampicillin administered as IAP

Table 2 provides details on eight out of the 15 neonates with severe disease who were clinically ill at birth. The median time to blood culture collection was 1 h (IQR 0.0–3.0, range 0–179.5), while the median time to first antibiotics was 2.0 h (IQR 1.0–5.0, range 0–20). One infant at risk died despite receiving immediate antibiotics at birth due to an infection caused by multidrug-resistant *E. coli*. The NSC would have recommended immediate antibiotics for all these eight neonates.

Table 3 details seven of the 15 neonates with severe disease who were initially well-appearing at birth. The median time between disease onset and blood culture collection was 1 h (IQR 0.0–5.8), and the median time for first antibiotic administration was 1.0 h (IQR 0.0–9.5). Three newborns (one assigned to the SCO programme and two under routine care) received antibiotics more than 1 h after disease onset. Only two (28.6%) of seven neonates were assigned to the SCO programme at birth because of risk factors. Among these seven newborns with severe disease and well-appearing at birth, five developed meningitis. Notably, one case involved a newborn who was initially well-appearing and had been exposed to adequate IAP (ampicillin administered > 24 h before delivery), but at 25 h of life developed the first signs of meningitis due to an ampicillin-resistant *E. coli* strain. The NSC would have assigned to routine care at birth all these seven neonates with severe disease who were initially well-appearing. Four of these infants developed first signs of illness > 24 h after birth, thus exceeding the time window considered by the NSC.

## Discussion

This is the first study examining how promptly culture-confirmed EOS cases are recognized and treated under the SCO strategy. The vast majority (79%) of infants clinically ill at birth presented with “major” clinical signs, which under the SCO program should trigger immediate blood culture and antibiotic treatment. Retrospective NSC calculation would have recommended antibiotics at 4 h of life in a similar proportion of cases (88%). However, in our cohort, a number of these cases escaped early identification and received antibiotics > 1 h after birth. This finding highlights an implementation gap rather than a limitation of the SCO algorithm itself: even though the protocol correctly identifies “major” signs as requiring immediate intervention, real-world adherence may be suboptimal [34, 35]. This gap underscore the importance of ongoing staff training and periodic retraining to optimize protocol adherence, as sepsis education programs have shown to improve care processes and patient outcomes [36].

By contrast, neonates with EOS who were initially well-appearing at birth were inadequately identified using the SCO strategy (~ 60% were assigned to routine care at birth and the median time from disease onset to antibiotics was 6 h). Many of these cases—including those with severe disease—occurred in infants without identifiable risk factors (approximately 40% of all EOS cases lacked risk factors). Clinicians may underestimate early signs of illness in newborns who are well-appearing at birth because they lack risk factors. Also, as shown in our cohort, clinicians may be falsely reassured by adequate IAP, although extended IAP courses can fail if the pathogen (e.g. *E. coli)* is resistant to the administered antibiotic [37]. Similarly, the secondary analysis—based on retrospective application of NSC—showed that most of initially well-appearing infants (~ 70%) would have been assigned to routine care. However, NSC performance improves when all neonates, including those initially assigned to routine care, undergo continued clinical evaluation for at least the first 24 h of life. Consistent with findings from a meta-analysis and recent reports [23, 28], in our cohort, 47% of EOS cases would have been assigned to antibiotic therapy by the NSC at 4 h of life, increasing to 58% when clinical reassessment was extended to 24 h of life. However, the intervals recommended by the NSC for clinical reassessments within the first 24 h may be too prolonged, potentially delaying timely identification of EOS. Furthermore, by restricting clinical observation to the first 24 postnatal hours, NSC application in our cohort would have potentially missed approximately one-third of neonates who developed symptoms after birth, consistent with the previous literature [38]. Since a considerable proportion of EOS cases lack both risk factors and clinical signs at birth [39], timely detection requires either extending the SCO to all newborns regardless of risk factors (so-called universal SCO strategy) [1, 40], or integrating NSC at birth with universal SCO during the subsequent 48 h [41]. However, we acknowledge that in many healthcare settings, early discharge policies may preclude prolonged in-hospital observation. In such settings, clinicians and families should be aware that EOS may present at home, and appropriate education regarding warning signs and access to prompt medical evaluation should be provided.

Our study highlights that the distinction between “major” and “minor” signs may be a reliable guide in the management of infected neonates. We acknowledge that without a control group of healthy infants, we cannot determine which clinical signs allow identification of infected versus non-infected neonates. However, our study design allows us to describe symptom patterns among confirmed cases of EOS, and their association with disease severity: (1) all neonates who developed severe disease presented with at least one “major” sign at disease onset, which should have triggered immediate blood culture collection and antibiotics [27]; (2) no infant who initially presented with isolated respiratory signs subsequently developed severe disease; (3) subtle neurological signs (e.g. hypotonia, irritability) were common at the onset of severe EOS. Notably, hypotonia -not included among signs of illness listed by either SCO or NSC-was commonly observed at disease onset in severe cases. These findings are consistent with a recent systematic review and meta-analysis [42] demonstrating that in young infants aged 0–59 days, fast breathing was not significantly associated with sepsis, while neurological signs such as drowsiness were among the strongest predictors of confirmed sepsis and mortality.

Finally, 12% of EOS cases remained asymptomatic, with less than half receiving antibiotics at birth. Although GBS and *E. coli* are generally not considered contaminants, distinguishing true EOS cases from transient bacteraemia in asymptomatic neonates remains challenging, and the clinical benefit of treating such cases remains uncertain [33].

This is the first study evaluating the real-world performance of the SCO program in culture-confirmed EOS cases. Additional strengths include the prospective, multicentre design and the relatively high number of culture-confirmed cases within a population-based surveillance framework.

Our study has several limitations. First, it is an observational, non-randomized study. According to the SCO protocol, vital signs in neonates at risk were monitored prospectively, as documented in the SCO standardized form; however, data may have been incomplete or unavailable in neonates assigned to routine care, who did not receive systematic surveillance. This creates a potential detection and timing bias: infants assigned to routine care may have had subtle clinical signs present earlier than documented, with later documented onset reflecting delayed recognition rather than true later disease onset. Second, the NSC score calculation was performed retrospectively, introducing potential bias, and how promptly the NSC would have recommended antibiotic administration in real-world practice cannot be concluded. However, since NSC calculation is based on objective data, the risk of bias may be minimal. Third, neonates developing clinical signs of illness after 48 h of life may represent nosocomial infections [43] and, by definition, cannot be identified by any EOS risk stratification strategy [33]. However, excluding these cases would be inconsistent with the epidemiological definition of EOS adopted by major surveillance networks which define EOS as infection occurring within the first 72 h of life [2, 8] and would underestimate the clinical burden that management strategies must address. Also, our analysis is confined to the two pathogens included in our surveillance—GBS and *E. coli*—that, in our region [24] and in countries with established antenatal GBS screening programs [2, 44] account for approximately 70% of EOS cases. Although 57 culture-proven EOS cases may appear limited, this number is consistent with the expected incidence of EOS in our population [24], in keeping with high-income countries [2, 45]. This sample size does not provide sufficient statistical power for meaningful multivariable modelling or stratified subgroup analyses, and the results should be interpreted with caution. Finally, we included only culture-proven EOS cases; therefore, our study represents a descriptive analysis of recognition and treatment timing within a network adopting the SCO approach, with retrospective modelling of NSC recommendations. Consequently, it does not allow for assessment of the overall performance of these strategies, including specificity, positive or negative predictive values. However, this methodological approach is consistent with previous studies comparing the sensitivity of different EOS management strategies using culture-positive cohorts [28]. The specificity of SCO has been evaluated in previous population-based studies from our region, which demonstrated that SCO reduces unnecessary antibiotic exposure of healthy uninfected infants [31].

## Conclusions

Both SCO and NSC strategies rely upon an initial EOS risk assessment based on maternal risk factors, and then adjust it based on clinical findings. Their implementation has considerably reduced unwarranted antibiotic exposure of healthy uninfected infants. However, in our cohort of EOS cases, one-third had neither maternal risk factors nor clinical signs of illness at birth, and under the SCO strategy were assigned to routine care. Similarly, retrospective modelling of NSC recommendations showed that a substantial proportion of initially well-appearing infected neonates would have been assigned to routine care. Also, some treatment delays were not consistent with SCO protocol recommendations, highlighting a gap between guideline quality and real-world implementation that underscores the need for ongoing staff training and periodic retraining. To ensure timely recognition without increasing unwarranted antibiotics, close observation of all infants over the first 24–48 h after birth may be beneficial, where resources and clinical setting allow. In culture-confirmed cases, management may be optimized by recognizing symptom patterns that predict severe disease: isolated respiratory signs are not predictive of subsequent severe disease, whereas “major” signs or subtle neurological signs (i.e. hypotonia and irritability) are frequently observed at the onset of severe EOS.

## Supplementary Information

Below is the link to the electronic supplementary material.

ESM 1 (PDF 126 KB)

## Data Availability

Individual participant data that underlie the results reported in this article, after de-identification will be made available beginning 5 months and ending 36 months after article publication to researchers who make a methodologically sound proposal. Proposals should be directed to 79,638@studenti.unimore.it. To gain access, data requestors will need to sign a data access agreement.

## Abbreviations

AAP: American Academy of Pediatrics
EOS: Early-Onset Sepsis
GBS: Group B Streptococcus
LBs: Live Births
NSC: Neonatal Sepsis risk Calculator

## Declarations

### Authoring group for the prevention of Group B Streptococcus neonatal infections, Emilia Romagna

M. De Angelis, S. Spinedi, S. Ambretti, M.G. Capretti, L. Corvaglia, M De Angelis, A. Dondi, M. Lanari, L. Pasini, L. Ragni, C. Vocale (Bologna); L. Marrozzini, F Torcetta (Carpi); E. Di Grande, A. Polese (Castelnuovo Monti); V. Rizzo, M. Stella, L. Di Luca (Cesena); A. Zucchini (Faenza); M. Azzalli, S. Fanaro, A. Solinas (Ferrara); P. Bacchini, M.L. Bidetti (Fidenza); R.M. Pulvirenti, E. Valletta, (Forlì); C. Alessandrini, S. Storchi Incerti (Guastalla); C. Di Carlo, A. Lanzoni, D. Silvestrini (Imola); A. Berardi, F. Ferrari, L. Lugli, C. Venturelli, M Sarti (Modena); A. Volta (Montecchio Emilia); I. Dodi, L. Gambini, S. Perrone (Parma); B. Guidi (Pavullo); B. Benenati, G. Biasucci, N. De Paulis, D. Padrini, S. Riboni (Piacenza); M. Cricca, V. Sambri (Pievesestina); L. Casadio, G. Piccinini, F. Marchetti (Ravenna); L. Baroni, E. Carretto, V. Fiorini, S. Fornaciari, G. Gargano, S. Pedori, C. Zuelli (Reggio Emilia); G. Ancora, S. Bolognesi, MC. China, I. Papa, F. Nanni, G. Vergine, D. Scarponi (Rimini); R. Pagano, C. Rota (Sassuolo).

### Disclaimer

The funders had no role in the design and conduct of the study.
