# Correction to: Real-world use of Serial Clinical Observation in culture-proven early-onset sepsis: timing of recognition, treatment and retrospective comparison with the Neonatal Sepsis Calculator

**DOI:** 10.1007/s00431-026-07005-2

**Published:** 2026-05-08

**Authors:** Francesca Miselli, Licia Lugli, Giorgia Dragone, Luca Bedetti, Sofia Spinedi, Mariagrazia Capretti, Lucia Marrozzini, Luisa Di Luca, Silvia Fanaro, Lucia Gambini, Giacomo Biasucci, Giancarlo Piccinini, Davide Scarponi, Irene Papa, Francesca Nanni, Lorenza Baroni, Rossella Pagano, Valeria Capone, Alberto Berardi

**Affiliations:** 1 Neonatal Intensive Care Unit, University Hospital of Modena, Via del Pozzo, 41124 Modena, Italy; 2 PhD Program in Clinical and Experimental Medicine, University of Modena and Reggio Emilia, Via Università 4, 41121 Modena, Italy; 3 Postgraduate Training in Obstetrics and Gynecology, University of Torino, Via Verdi 8, 10124 Turin, Italy; 4 Maggiore University Hospital, Largo Bartolo Nigrisoli 2, 40133 Bologna, Italy; 5 S. Orsola-Malpighi University Hospital, Via Giuseppe Massarenti 9, 40138 Bologna, Italy; 6 Ramazzini Hospital of Carpi, Via Giovanni Rodolfo Baroni 1, 41012 Carpi, Modena Italy; 7 Bufalini Hospital, Viale Ghirotti 286, 47521 Cesena, Italy; 8 Cona University Hospital, Via Aldo Moro 8, 44124 Cona, Ferrara Italy; 9 University Hospital, Via Gramsci 14, 43126 Parma, Italy; 10 Guglielmo da Saliceto Hospital, Via Taverna 49, 29121 Piacenza, Italy; 11 S. Maria Delle Croci Hospital, Viale Randi 5, 48121 Ravenna, Italy; 12 Infermi Hospital, Viale Luigi Settembrini 2, 47923 Rimini, Italy; 13 Santa Maria Nuova Hospital, Viale Risorgimento 80, 42123 Reggio Emilia, Italy; 14 Hospital of Sassuolo, Via Francesco Ruini 2, 41049 Sassuolo, Modena Italy

**Correction to: European Journal of Pediatrics (2026) 185:274**

10.1007/s00431-026-06905-7

In the original publication of the article “Real-world use of Serial Clinical Observation in culture-proven early-onset sepsis: timing of recognition, treatment and retrospective comparison with the Neonatal Sepsis Calculator” (DOI: 10.1007/s00431-026-06905-7), the legend for Figure 1 was inadvertently omitted.

**Published version:**

The article was originally published with Figure 1 lacking its explanatory legend.
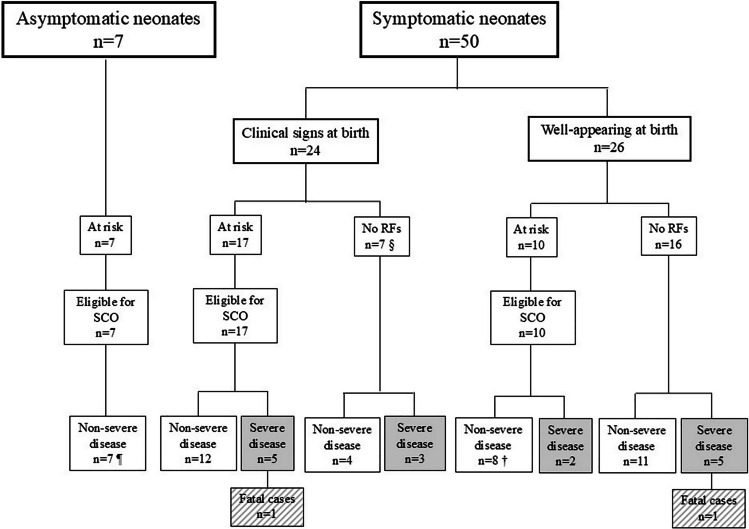


**Corrected form:**

The legend for Figure 1 should have been included as follows:
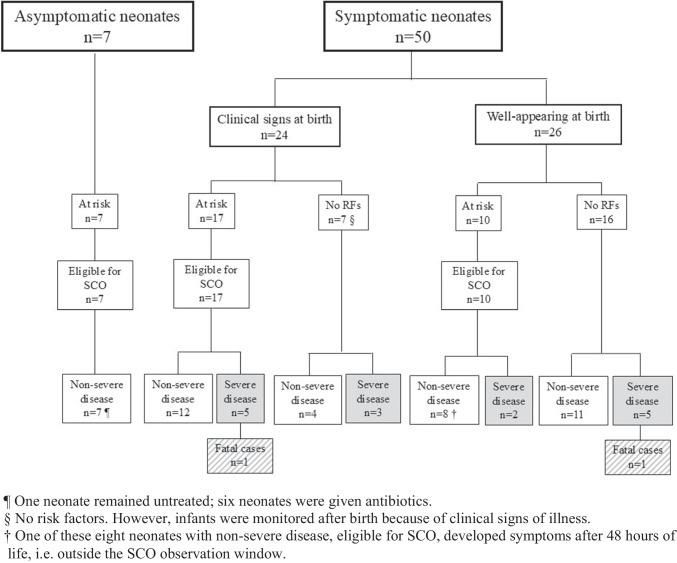


¶ One neonate remained untreated; six neonates were given antibiotics.

§ No risk factors. However, infants were monitored after birth because of clinical signs of illness.

† One of these eight neonates with non-severe disease, eligible for SCO, developed symptoms after 48 hours of life, i.e. outside the SCO observation window.

The original article has been corrected.

