# Supplementary material for: Real-world use of Serial Clinical Observation in culture-proven early-onset sepsis: timing of recognition, treatment and retrospective comparison with the Neonatal Sepsis Calculator
Source: Eur J Pediatr. 2026 Apr 16;185(5):274. doi: 10.1007/s00431-026-06905-7 (PMC13086652; doi:10.1007/s00431-026-06905-7)
Supplement: Supplementary file 1 — (PDF 126 KB) [file 431_2026_6905_MOESM1_ESM.pdf]

Supplementary Information

Online Resource 1

List of clinical signs that are required in the Case Reporting Form for each case of confirmed Early-Onset Sepsis (EOS). The physician completing the EOS form is explicitly required to document all symptoms (Yes/No/Unknown) present at the time of disease onset, as well as, separately, those that appeared after onset.

Body temperature (°C) and temperature measurement site

Respiratory rate (breaths/min)

Heart rate (beats/min)

Skin colour

Consciousness

Bulging anterior fontanelle

Toxic appearance

Fever > 38°C

Hypothermia < 36°C

Hypotension (< 5th percentile)

Decreased urine output

Hypotonia

Lethargy

Irritability

Seizures

Apneas

Capillary refill time > 3 seconds

Feeding difficulties

Abdominal distension

Vomiting

Diarrhea

Jaundice

Online Resource 2

Clinical criteria triggering blood culture and antibiotics for suspected Early-Onset Sepsis according to Serial Clinical Observation management, divided into “major” and “minor” signs. Blood culture and antibiotics should be carried out i) immediately in the presence of major signs or ii) when minor signs worsen or persist for more than 12–24 hours. However, the decision to perform a blood culture and initiate empirical antibiotic therapy ultimately remains at the clinician's discretion. Modified with permission from: 28. Berardi A, Zinani I, Bedetti L, et al. Should we give antibiotics to neonates with mild non-progressive symptoms? A comparison of serial clinical observation and the neonatal sepsis risk calculator. Front Pediatr 2022; 10. https://www.frontiersin.org/articles/10.3389/fped.2022.882416.

**Minor ‡**	**Major**
Tachypnoea (> 60/minute) without increased respiratory effort	Moderate-to-severe respiratory distress (i.e. tachypnoea *plus* increased respiratory effort) requiring respiratory support §
Tachycardia > 160 beats/minute	Hypoxia, reduced O_2 saturation
Metabolic acidosis (BE ≤ - 10 mmol/l)	Reduced skin perfusion<br>Refill time ≥3 seconds<br>Signs of shock
Temperature <36°C or > 37.5° and <38 °C	Temperature ≥ 38 °C*
	Worsening of the general condition<br>Apnoea<br>Lethargy<br>Irritability<br>Seizures
	Greyish, pallor or marbling of the skin colour

‡ Laboratory evaluation can be delayed (on the basis of the clinician's judgment) in the presence of minor and non-progressive symptoms in the first 12-24 hours of life. In the case of mild symptoms onset, babies are re-checked at 2-hour intervals

§ Respiratory support includes positive pressure ventilation outside of the delivery room

* Outside of the delivery room
